# The Regulatory Effects of Traditional Chinese Medicine on Ferroptosis

**DOI:** 10.1155/2022/4578381

**Published:** 2022-09-19

**Authors:** Qian Gao, Xue-dong Yin, Fan Zhang, Yi-Zhun Zhu, Zhi-ling Li

**Affiliations:** ^1^Department of Pharmacy, Shanghai Children's Hospital, School of Medicine, Shanghai Jiao Tong University, Shanghai 200062, China; ^2^School of Medicine, Shanghai Jiao Tong University, Shanghai 200125, China; ^3^Department of Pharmacy, Peking Union Medical College Hospital, Chinese Academy of Medical Sciences & Peking Union Medical College, Beijing 100730, China; ^4^State Key Laboratory of Quality Research in Chinese Medicine & School of Pharmacy, Macau University of Science and Technology, Taipa, China; ^5^Shanghai Key Laboratory of Bioactive Small Molecules & School of Pharmacy, Fudan University, Shanghai, China

## Abstract

Traditional Chinese medicine (TCM) has significantly contributed to protecting human health and promoting the progress of world civilization. A total of 2,711 TCMs are included in the 2020 version of the Chinese Pharmacopoeia, which is an integral part of the world's medical resources. Tu Youyou and her team discovered and purified artemisinin. And their contributions made the values and advantageous effects of TCM more and more recognized by the international community. There has been a lot of studies on TCM to treat diseases through antioxidant mechanisms, the reports on the new mechanisms beyond antioxidants of TCM has also increased year by year. Recently, many TCMs appear to have significant effects in regulating ferroptosis. Ferroptosis is an iron-dependent, non-apoptotic, regulated cell death characterized by intracellular lipid peroxide accumulation and oxidative membrane damage. Recently, accumulating studies have demonstrated that numerous organ injuries and pathophysiological process of many diseases are companied with ferroptosis, such as cancer, neurodegenerative disease, acute renal injury, arteriosclerosis, diabetes, and ischemia-reperfusion injury. This work mainly introduces dozens of TCMs that can regulate ferroptosis and their possible mechanisms and targets.

## 1. Introduction

The history of traditional Chinese medicine (TCM) has a long history, and written records of TCM have existed for more than 5,000 years. TCM reflects the wisdom of the Chinese people, which has evolved for thousands of years. It contains the precious experience of ancient Chinese people in fighting against diseases. TCM is one of the world's oldest medical systems, a healing approach based on the foundation of Chinese philosophy that utilizes the balance between mankind and nature. Its theories include the concept of yin and yang, the Five Elements, zang-fu, channels-collaterals, qi, blood, body fluid, methods of diagnosis, and the differentiation of symptom-complexes [1]. TCM is characterized by two primary features: a holistic treatment strategy based on the differentiation of syndromes. The selection of TCM natural products is guided by their taste and Yin and Yang qualities [2]. TCM contributes greatly to the medical field by providing active pharmaceutical ingredient [3, 4]. TCM functions by individual adjustment of multiple components and targets and facilitates the transformation of the body from an abnormal to a normal state.

Ferroptosis is a novel form of programmed non-apoptotic cell death and is characterized by intracellular iron-dependent lipid peroxidation and accumulation of reactive oxygen species (ROS). The conception of ferroptosis was proposed by Dixon et al. in 2012, and the discovery of ferroptosis emerged from their identification in 2003 of erastin that induced a non-apoptotic form of cell death [5]. Iron ions are not only importance trace elements in body but also are the triggered factor involved in the occurrence of ferroptosis [6]. Excessive Iron may directly generate plenty of ROS to induce oxidative damage and ferroptosis through the Fenton reaction [4]. Ferroptosis is involved in multiple diseases including cancer, arteriosclerosis, ischemia-reperfusion injury, neurodegenerative disease, and acute renal failure [7–12]. Glutathione peroxidase 4 (GPX4), ferroptosis suppressor protein 1 (FSP1), and dihydroorotate dehydrogenase (DHODH) constitute the three primary defense mechanisms against ferroptosis. They are as follows: 1) GPX4 specifically catalyzes lipid peroxidation to inactivate its oxidative activity in a GSH-dependent manner and protects the cells from ferroptosis [12–14]. 2) FSP1 is a GSH-independent ferroptosis inhibitor that acts as a redox catalyst, reducing coenzyme Q10 (CoQ10) to ubiquinol (CoQ10H2) at the cell membrane. CoQ10H2 acts as a lipophilic antioxidant by capturing free radicals and inhibiting lipid peroxides [15, 16]. 3) DHODH inhibits ferroptosis by inhibiting lipid peroxidation in mitochondria [17]. RSL3 and erastin are two experimental compounds that can induce ferroptosis. Dixon et al. found that erastin induced ferroptosis by blocking uptake of cystine through system Xc^−^, a cystine-glutamate anti-porter that transports extracellular cysteine into for the synthesis of GSH., resulting in depletion of GSH and cystine [18], and RSL3 inhibited GPX4 [13]. Lipid peroxidation is a free radical-driven reaction, which mainly affects metabolism of polyunsaturated fatty acids (PUFAs) in the cell membrane [19]. All mammalian cells have certain levels of PUFAs, and PUFAs is converted to phospholipid hydroperoxides (PLOOH). The products of lipid peroxidation include the PLOOH and malondialdehyde (MDA). During the ferroptosis process, ROS and phospholipid hydroperoxides (PLOOH) accumulate and cannot be neutralized efficiently. Excessive ROS may disrupt the integrity of the plasma membrane [20].

Ferroptosis is not only associated with several diseases but is also a key component of many signaling pathways. The process of ferroptosis can also be targeted by drugs. In this work, we mainly focused on regulating ferroptosis by TCMs and their active pharmaceutical ingredient. We reviewed the primary mechanisms of how TCMs affect ferroptosis. It is reported that dysregulation of ferroptosis is linked with numerous physiological conditions and pathological stress. However, ferroptosis is a two-edged sword. On the one hand, it induces the non-apoptotic destruction of cancer cells [21]. but on the other hand, it may lead to organ damage [12, 22, 23]. Among TCMs, some active pharmaceutical ingredients are ferroptosis inducers and have excellent anticancer efficacy, while some show organ-protective effects by acting as ferroptosis inhibitors.

## 2. The Main TCMs for Regulating Ferroptosis

### 2.1. Artemisinin

Artemisinin, as a natural sesquiterpene lactone compound, was isolated for the first time from the Asteraceae plant Artemisia arbusus in 1971 by the Chinese scientist Tu Youyou who won the Nobel Prize in Medicine. Artemisinin is now widely used in the treatment of malaria [24, 25]. Dihydroartemisinin (DHA), a derivative of artemisinin, also has therapeutic effects on various cancers, including liver cancer [26], breast cancer [27], and lung cancer [28]. DHA functions by inhibiting the PRIM2/SLC7A11 axis and induces ferroptosis and inhibits cell proliferation. The peroxide bridge structure in the molecular structure of DHA may cause ferroptosis in tumor cells by disrupting the intracellular redox equilibrium [29–31]. Lin et al. found that DHA inhibits tumor progression in head and neck carcinoma by inducing ferroptosis in tumor cells [32]. Artemisinin and its derivatives, as the basic drugs for malaria treatment, save the lives of hundreds of thousands of patients around the world every year. Recently, discoveries in oncology studies have shown that artemisinin and its derivatives act as ferroptosis inducers by inducing tumor cells ferroptosis to exert anticancer effects. So far, artemisinin and its derivatives have no obvious serious adverse reactions using antimalarial treatment, and have very high safety. However, it has been reported that when artesunate suppositories are administered rectally, about 6% of patients experience tenesmus, but serious adverse reactions such as neutropenia are rare [33, 34].

### 2.2. Leonurine (Also Named as SCM-198)

Leonurine is a primary active alkaloid compound extracted and purified from Motherwort, a traditional Chinese herbal medicine [35]. Motherwort has the effects of promoting blood circulation and regulating menstruation, diuresis and swelling, clearing heat and detoxification, and is often used for blood stagnation, amenorrhea and dysmenorrhea. In the past, motherwort was considered as a safe and non-toxic TCM. In recent years, accumulating studies have found that long-term and high-dose use of motherwort will cause some adverse reactions, mainly manifested as sudden feeling of general weakness, paralysis of the lower limbs, severe sweating, decreased blood pressure, and rapid breathing, and even cause uterine contractions and lead to miscarriage in pregnant women [36, 37].

Leonurine reduces renal podocyte injury, lipopolysaccharide-induced acute kidney injury, and renal fibrosis by inhibiting oxidative stress via reducing the production of ROS and protecting the kidneys [38, 39]. Ferroptosis is mediated by phospholipid peroxidation, a process that requires ROS and transition metal iron. Recently, Leonurine has been shown to inhibit ferroptosis by activating the antioxidant transcription factor Nrf2 and play a protective role against liver [40] and kidney [41] injuries. In addition, leonurine has also been confirmed to inhibit the expression of fibronectin by blocking the transforming growth factor-*β* (TGF-*β*)/NF-*κ*B signaling pathway and in chronic kidney disease rat model, leonurine can reduce tubulointerstitial fibrosis caused by unilateral ureteral obstruction [38].

### 2.3. Curculigoside, Astragalus Polysaccharide and Astragaloside IV

Astragalus membranaceu is sweet in taste and warm in nature. It has the functions of invigorating qi and raising yang, strengthening the surface and relieving sweat, and diuresis and swelling. It is a commonly used medicine in TCM for the treatment of kidney disease. Clinical adverse reactions caused by it are rare, and the drug is relatively safe. Curculigoside (CUR) is a phenolic glycoside compound isolated and purified from Astragalus membranaceu. It shows various pharmacological activities, such as anti-inflammatory [42] and antioxidant [43], anti-osteoporotic effects [44]. Wang et al. found that CUR protects against hydrogen peroxide-induced osteoblast impairment by alleviating oxidative damage [45]. Another study has confirmed the efficacy of CUR in attenuating excess iron-induced bone loss and oxidative stress by inhibiting ROS production and increasing superoxide dismutase (SOD) and GPX4 [46]. The protective effect of CUR may be associated with the inhibition of ferroptosis. CUR inhibits intestinal epithelial cells ferroptosis by inducing GPX4 expression in ulcerative colitis (UC) [47].

Astragalus polysaccharide (APS), another bioactive compound extracted from Astragalus membranaceus, also possesses several potential pharmacological effects, including anti-inflammation [48], anti-infection [49], anti-tumor [50], and immune regulatory properties [51]. Similar to CUR, APS also protects intestinal epithelial cells (IECs) against ferroptosis by inhibiting Nrf2/HO-1(Heme oxygenase 1) signaling pathways and reduces lesions in a murine model of experimental colitis [52].

Astragaloside IV, another compound isolated from Astragalus membranaceus, protects the endothelial cells by decreasing ROS production and oxidative stress [53]. Sheng et al. suggested that Astragaloside IV may prevent myocardial damage by protecting the Human Umbilical Vein Endothelial Cells (HUVECs) against ferroptosis [54].

### 2.4. Quercetin, QCT

Quercetin (3,30,40,5,7-pentahydroxyflavone) is a unique bioactive natural flavonoid widely distributed in nature and abundant in different vegetables. QCT has a wide range of pharmacological effects, including anticancer [55], antioxidant [56], and anti-inflammatory [57] effects. The antitumor activity of QCT may be associated with its pro-apoptotic effect [47]. However, in contrast, low-dose QCT (10 mg/kg) also protects against diabetic kidney injury by inhibiting apoptosis [58] in hypercholesterolemic mice, it also alleviates acute kidney injury (AKI) by inhibiting the expression of activating transcription factor 3 (ATF3) and blocking ferroptosis in renal proximal tubular epithelial cells [59]. QCT also impedes the accumulation of iron, attenuates lipid peroxide, and restores the expression of the voltage-dependent anion-selective channel protein 2(VDAC2). It shows protective effects against type 2 diabetes by blocking ferroptosis in pancreatic *β* cells [60]. From these studies, we concluded that QCT might inhibit cancer progression by promoting apoptosis and exert a cytoprotective effect by inhibiting apoptosis and ferroptosis.

### 2.5. Erianin

Erianin, a natural product isolated from the herb Dendrobium, exerts many bioactive effects, including antitumor activity in several cancers. A study shows that erianin may be a promising medicine for osteosarcoma because it induces G2/M cell cycle arrest and triggers regulated cell death, such as apoptosis and autophagy [61]. Zhu et al. reported that erianin exhibits antitumor activity in bladder cancer cells by inducing mitochondrial apoptosis and the JNK pathway [62]. Liu et al. showed that erianin may be used for treating human nasopharyngeal carcinoma since it induces cell apoptosis through the ERK signaling pathway [63]. Furthermore, it has been reported that erianin shows antitumor activity in lung cancer cells by inducing ferroptosis and G2/M-phase arrest to inhibit cell proliferation and migration. This study, for the first time, also found that erianin induces ferroptosis via the Ca^2+^/CaM signaling pathway in lung cancer cells and inhibits the progression of the tumor [64]. We concluded that the antitumor effect of erianin is achieved by both pro-apoptotic and non-apoptotic pathways including ferroptosis.

### 2.6. Curcumin

Curcumin([1,7-bis(4-hydroxy-3-methoxypheny1)-l,6-heptadiene-3,5-dione]), discovered in Curcuma longa L., has been widely and safely consumed for hundreds of years. It is a natural food pigment and also shows potential applications in cancer treatment [65, 66]. Due to its rare adverse reactions [67], curcumin has been clinically used for the treatment of various diseases besides tumors, such as diabetes, obesity, cardiovascular disease, lung disease, neurological disease and autoimmune disease [68]. Previous studies have demonstrated that Curcumin inhibits the growth of cancer cells, including pancreatic cancer [69] and prostate cancer [70], by inhibiting cell proliferation and inducing cell apoptosis. Curcumin has been shown to be capable of eliminating ROS [71], once intracellular ROS accumulation significantly exceeds normal levels, it leads to significant damage to DNA, lipids, and proteins and finally induces cell death, including ferroptosis [4, 72]. A series of studies have shown that Curcumin inhibits cancer progression by inducing ferroptosis. Li et al. have found that Curcumin triggers ferroptosis in breast cancer cells by upregulating HO-1 expression [73]. Zhang et al. verified that curcumenol, another antitumor component extracted from turmeric thizomes, induces ferroptosis in lung cancer cells through the lncRNA H19/miR-19b-3p/FTH1 axis [74]. Curcumin has the potential to be a broad-spectrum and safe anticancer drug.

### 2.7. Epigallocatechin-3-Gallate, EGCG

EGCG, an active polyphenol compound found in green tea, belongs to the same polyphenol family as curcumin [75] and shows multiple biological activities, including antitumor, anti-inflammatory, anti-bacterial, and cytoprotective activities [76, 77]. EGCG possesses powerful antioxidant activity due to its special stereochemical structure. It penetrates the blood-brain barrier and chelates Fe^3+^ to decrease ROS and *α*-synuclein, thereby preventing and treating neurodegeneration [78]. EGCG also regulates the expression of AMP-activated protein kinase (AMPK) and reduces lipid accumulation in canine hepatocytes [79]. Studies showed that EGCG protects cardiomyocytes against DOX-induced cardiotoxicity by inhibiting apoptosis and ferroptosis in Sarcoma 180 tumor-bearing mice [80, 81]. Furthermore, EGCG also ameliorates ionizing radiation-induced ferroptosis in mouse intestinal epithelial cells by deregulating ROS and activating Nrf2 and its downstream antioxidant proteins, including SLC7A11, HO-1, GPX4 [82]. It also alleviates erastin-induced ferroptosis in pancreatic Beta-cells [75]. EGCG also inhibits ferroptosis in cerebellar granule neurons by promoting PKD1 phosphorylation after spinal injury [83].

### 2.8. Glycyrrhiza

The traditional herbal medicine licorice, the dried rhizome of Glycyrrhiza (GL), is used for spleen and stomach weakness, fatigue and fatigue, palpitations and shortness of breath, cough, and phlegm. Adverse reactions of GL and its derivatives includes increased blood pressure, edema, abdominal pain and so on. In addition, pregnant women should also use it with caution [84]. GL extracted from Glycyrrhiza uralensis Fishch is a natural glycosyl triterpenoid and is identified as an inhibitor of the high-mobility group box1 (HMGB1). GL has a neuroprotective effect on brain injury by inhibiting HMGB1 and its downstream inflammatory factors and reducing oxidative stress [85]. Recent studies have shown that cell ferroptosis induces HMGB1 release and inflammation in the acute pancreatitis model [86]. It has been shown that HMGB1 is released from injured cells and shows cytokine activity that has been linked with the pathogenesis of many central nervous system (CNS) diseases, including neonatal hypoxic-ischemic brain damage (HIBD) [87]. Therefore, as an HMGB1 inhibitor, GL provides a potential treatment for HIBD, and GL has been shown to reduce ferroptosis-mediated brain damage by modulating the GPX4 axis [88]. Echinatin, another active ingredient of licorice, effectively suppresses the activation of the NLRP3 inflammasome by targeting HSP90 and is used for treating a variety of human inflammatory diseases [89].

### 2.9. Honokiol, HNK

HNK is a biphenolic compound extracted from various parts of Magnolia officinalis [90]. HNK shows efficient and specific antitumor effects. HNK has been reported to suppress the growth of cancer cells by inducing apoptosis and autophagy. HNK promotes ROS generation and induces ROS-mediated cell death by regulating p53/PI3K/Akt/mTOR signaling pathway [91–93]. Recently, HNK also was shown to increase the intracellular ROS level by decreasing the activity of GPX4, thereby killing colon cancer cells (CCCs) [94]. Therefore, HNK shows potential in treating solid tumors. Lai et al. reported that HNK induces acute myeloid leukemia (AML) cells ferroptosis by upregulating HMOX1 [95].

### 2.10. Tanshinone IIA

Salvia miltiorrhiza Bunge. is a perennial upright herb of the genus Sage in the family Dicotyledonaceae; TCM salvia is a dried root and rhizome, which activates blood and dispels stasis and clears the heart [96]. With the increase in clinical application, reports of its toxic and side effects are also increasing, including allergic skin rash, liver damage, and gastrointestinal bleeding [97]. The main bioactive ingredients of salvia include liposoluble salvia ketone and water-soluble salvia phenolic acids [98]. Tanshinone IIA (Tan IIA) was isolated from Salvia miltiorrhiza Bunge. and found to exert an antineoplastic effect in gastric cancer cells MKN-45 by inducing apoptosis and cell cycle arrest [99] and in AGS cells by suppressing insulin-like growth factor receptor (IGFR), epidermal growth factor receptor (EGFR) expression and blocking PI3K/Akt/mTOR pathways [100, 101]. A study showed that Tan IIA upregulates the expression of p53 and p53 is recruited to the SCL7A11 promotor to block the transcription of SLC7A11, which encodes system Xc^−^, a cystine-glutamate anti-porter that transports extracellular cysteine into for the synthesis of GSH. Consequently, Tan IIA induces ROS-mediated ferroptosis [102, 103].

### 2.11. 1,6-O-O-Diacetyl-Britannilactone (OABL)

Sesquiterpene lactones (STLs), are a lead bioactive component extracted from Inula japonica Thunb. They have been used for the treatment of multiple inflammatory diseases, including fever, migraine, arthritis, and atherosclerosis with few mild side effects [104, 105]. 1,6-o-o-diacetyl-britannilactone (OABL) is another STL mainly extracted from Inula Britannica L. OABL suppresses NO and PGE2 synthesis in RAW264.7 macrophages to exert an anti-inflammatory effect [106] and antineoplastic effect [107]. Oxidative stress and uncontrolled neuroinflammation induce neuronal damage in neurodegenerative diseases, such as Alzheimer's disease (AD) [108]. OABL may treat neurodegenerative disease by decreasing amyloid plaques (deposits of A*β*) and neurofibrillary tangles (hyperphosphorylated Tau). OABL increased the GSH level and reduced the MDA level in 5xFAD mice, showing that OABL protects neurons by inhibiting ferroptosis [109].

### 2.12. Beta-Elemene

Beta-elemene is a Class II anticancer drug extracted and purified from TCM curcumae rhizome. Beta-elemene has been used to treat various cancers, including colorectal cancer [110, 111]. Ferroptosis inhibitors prevented cell death mediated by beta-elemene treatment. This suggested that beta-elemene may inhibit tumor growth by inducing ferroptosis in KRAS mutant colorectal cancer cells. This study also has found that *β*-elemene also inhibits epithelial-mesenchymal transformation (EMT) by inhibiting metastasis of KRAS-mutant colon cancer tumor cells [112]. As a new ferroptosis inducer, beta-elemene is widely used in the treatment of various cancers, including lung, liver, brain, breast, ovary, gastric, and prostate cancers due to its low toxicity [113].

In addition to the above drugs, a variety of TCMs have also been found to be associated with cell ferroptosis.

### 2.13. Others

Ajuga nipponensis has abundant bioactivities, including hypoglycaemic [114] and antioxidant and hepatoprotective effects [115]. Recently, ajudecunoid C (ADC), a neoclerodane diterpenoid extracted from Ajuga nipponensis, has been shown to protect neurons from ferroptosis by activating the Nrf2 antioxidant pathway [116]. Cucurbitacins are a group of natural tetracyclic triterpenoids from oriental herbs, among which cucurbitacin B(CuB) is one of the most abundant and richly studied cucurbitacins extractive [117]. CuB mediates its potent antitumor activity not only by inducing cell apoptosis and related pathways [118] but also by the induction of a nonapoptotic pathway - CNE1 ferroptosis. CuB helps in the accumulation of iron and GSH depletion, resulting in the production of lipid peroxides. CuB also downregulates the expression of GPX4 [119]. Xie et al. have found that Selaginella extractive induces ferroptosis in breast cancer cells (MCF7) by enhancing the expression of VDAC2 channels and inhibiting the expression of Nedd4 E3 ubiquitin ligase, leading to the accumulation of peroxidation and the production of ROS [120]. Gao et al. have reported that Actinidia chinensis planch prevents gastric cancer by inducing apoptosis and ferroptosis in gastric cells [121]. Zhang et al. showed that Bufotalin accelerates lipid peroxidation by inducing degradation of GPX4, and treats human non-small cell lung cancer (NSCLC) by targeting ferroptosis [122]. It has been indicated that brucine is a weakly alkaline compound isolated from the seeds of Strychnosnux-vomica, and induces the accumulation of H_2_O_2_, causing ferroptosis in glioma cells by upregulating ATF3 [123]. Baicalein, a flavonoid compound mainly derived from the root of Scutellaria baicalensis, has recently been found as a ferroptosis inhibitor and functions through reducing iron accumulation, glutathione depletion, and lipid depletion peroxidation [124]. Brusatol, a natural product isolated from the Brucea javanica Merr., also regulates the ferroptosis process by blocking Nrf2 signaling [125]. So, as an inhibitor of Nrf2, brusatol showed therapeutic efficacy against human NSCLC by inducing ferroptosis through the FOCAD-FAK signaling pathway [126]. Ji et al. found that niujiaodihuang detoxify decoction, as an ferroptosis inhibitor, could alleviate injury by promoting GSH synthesis and enhance GPX4 activity in acute liver failure models [127]. Another study showed that realgar could induce ferroptosis by downregulating expression of Slc7A11 and Gpx4 in HK-2 cells to play a nephrotoxic effect, and the toxicity of realgar on HK-2 was dose-dependent [128]. Huang et al. reported that hedyotis diffusa injection could induce ferroptosis via the Bax/Bcl2/VDAC2/3 axis to inhibit the viability of lung adenocarcinoma cells [129]. Red ginseng polysaccharide, an active component of the herb *Panax ginseng* C. A. Meyer (Araliaceae), have an anticancer role in human lung cancr and breast cancer by inducing ferroptosis via targeting GPX4 [130].

In summary, tremendous endeavors have been made to explore the regulatory mechanisms of TCMs on ferroptosis over the past years.  Table 1 lists the Chinese and English names of representative TCMs which act as ferroptosis regulators.

## 3. Discussion and Outlook

**Figure 1:**
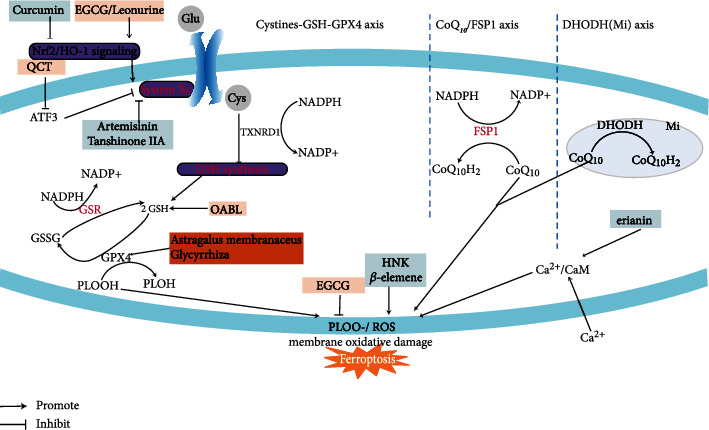
Significant TCMs involved in ferroptosis and their targets. There are three main iron death pathways, including the Cystine-GSH-GPX4 axis, CoQ10/FSP1, and DHODH axis. The frame of this color “peach” is filled with TCMs that are ferroptosis inhibitors; the frame of “light blue” is filled with ferroptosis inducers.

Here, we have summarized dozens of TCMs which regulate ferroptosis and have outlined their mechanisms and targets. DHA [28] and Tan IIA [102] induce ferroptosis by inhibiting System Xc^−^, while QCT [59] activates System Xc^−^ and blocks ferroptosis through the ATF3 signaling pathway. Curcumin [73] promotes ferroptosis by inhibiting Nrf-2 nuclear translocation and its downstream signaling pathway and System Xc^−^, while EGCG [82] and leonurine [40, 41] inhibits ferroptosis by promoting the Nrf-2/HO-1 signaling pathway. OABL [109] attenuates ferroptosis by upregulating GSH levels. Astragalus membranaceus [47, 53] promotes GPX4 activity to inhibit ferroptosis, while Glycyrrhiza [88] inhibits GPX4 to promote ferroptosis. EGCG [82] and *β*-elemene [113] inhibit ferroptosis by down-regulating intracellular ROS levels. HNK [94] promotes ferroptosis by upregulating intracellular ROS levels, and erianin promotes ferroptosis by upregulating ROS through the Ca^2+^/CaM signaling pathway [64]. Additionally, in vivo and in vitro experiments have shown that CuB, Selaginella, Actinidia chinensis plant, bufotalin, brucine, brusatol, realgar, hedyotis diffusa injection and Red ginseng polysaccharide may be used as ferroptosis inducers. In contrast, ADC, Baicalein, niujiaodihuang detoxify decoction may be used as ferroptosis inhibitors. The primary Chinese medicines that regulate ferroptosis and their possible targets are shown in Figure 1 [14, 17, 131, 132].

**Figure 2:**
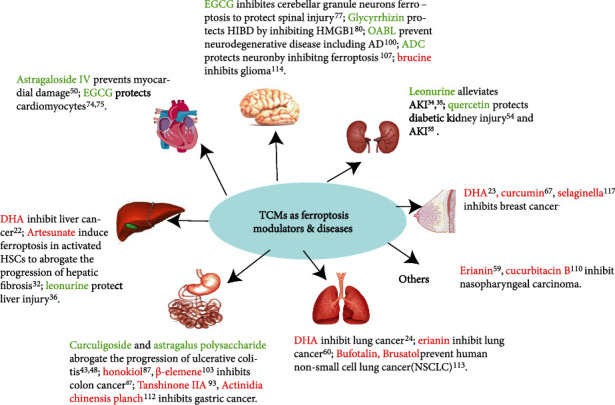
Ferroptosis has played an important role in multiple diseases, such as nervous system diseases, liver diseases, heart diseases, gastrointestinal diseases, lung diseases, kidney diseases, and breast diseases. TCM is engaged in the prevention and treatment of these diseases by regulating ferroptosis. Artemisinin derivatives:DHA, Artesunate; astragalus membranaceus derivatives: curculigoside, astragalus polysaccharide, astragaloside IV; glycyrrhiza derivatives: glycyrrhizin, echinatin; ajuga nipponensis: ajudecunoid C(ADC).

However, careful consideration should be given to the data obtained from cell experiments and animal experiments since cell and animal experiments are different from clinical investigations; future studies, especially clinical trials, are essential for using TCMs in ferroptosis-related diseases. These research studies will be complicated and challenging due to the complexity of TCMs components and the interaction of various therapeutic drugs in regulating ferroptosis-related diseases including cancers, auto-immune diseases. Nevertheless, TCMs have tremendous potential in the treatment of ferroptosis-related diseases in the future due to their low toxicity and side effects compared with chemical medicine. Compared with the classical ferroptosis inducers including erastin and RAS-selective lethal 3 (RSL3) the reported TCMs and their active ingredients that have a regulatory effect on ferroptosis have the characteristics of more regulatory targets, stable structure, high safety, low cost and easy availability, but the accumulation of related researches is insufficient and needs to be further explored. This study reviews the functions and possible targets of TCMs in regulating ferroptosis and provides theoretical support for the subsequent clinical application of TCMs in preventing and treating ferroptosis-related diseases (Figure 2).

## Figures and Tables

**Table 1:** Representative TCMs as Ferroptosis Regulators.

TCMs or active pharmaceutical ingredients	Function	Possible mechanism of regulating Ferroptosis	Herb Chinese name
Artemisinin/Dihydro-artemisinin	Inducer	Inhibited the function of system xc^−^	Qinghao
Leonurine	Inhibitor	Upregulated the Nrf2/HO-1 signaling	Yimucao
Astragalus Membranaceus	Inducer	Down-regulated the expression GPX4	Huangqi
Quercetin	Inhibitor	Activated system xc^−^ by inhibiting ATF3	Hupisu
Dendrobium/Erianin	Inducer	Upregulated the levels of intracellular ROS by Ca2^+^/CaM signaling pathway	Shihu
Curcuma longa L/curcumin	Inducer	Inhibited system xc^−^ by Nrf2/HO-1 signaling pathway	Jianghuang
Epigallocatechin-3-gallate,EGCG	Inhibitor	Activated system xc^−^ by inducing Nrf2 nucleus translocation	Biaomeishizi
Glycyrrhiza	Inhibitor	Upregulated the expression of GPX4	Gancao
Magnolia officinalis/Honokiol, HNK	Inducer	Upregulated the levels of ROS	Guangyulan
Salvia miltiorrhiza Bge./Tanshinone IIA	Inducer	Inhibited the function of system xc^−^	Danshen
Inula japonica Thunb./1,6-o-o-diacetyl-britannilactone, OABL	Inhibitor	Upregulated the levels of GSH	Xuanfuhua
Curcumae rhizome/Beta-elemene	Inducer	Upregulated the levels of ROS	Ezhu

The English and Chinese name of these TCMs comes from the Pharmacopoeia of the People's Republic of China (version 2020).

## Abbreviations

TCM: Traditional Chinese medicine
ROS: Reactive oxygen species
GPX4: Glutathione peroxidase 4
FSP1: Ferroptosis suppressor protein 1
DHODH: Dihydroorotate dehydrogenase
CoQ_10_: Coenzyme Q_10_
PLOOH: Phospholipid hydroperoxides
PUFA: Polyunsaturated fatty acyl
SOD: Superoxide dismutase
DHA: Dihydroartemisinin
TLR4: Toll-like receptor 4
Nrf2: Nuclear factor erythroid–related factor 2
HSCs: Hepatic stellate cells
CUR: Curculigoside
APS: Astragalus polysaccharide
IECs: Intestinal epithelial cells
QCT: Quercetin
AKI: Acute kidney injury
HIBD: Hypoxic-ischemic brain damage
ATF3: Activating transcription factor 3
VDAC2: Voltage-dependent anion-selective channel protein 2
HO-1: Heme oxygenase 1
GL: Glycyrrhizin
HMGB1: High-mobility group box1
HNK: Honokiol
OABL: 1,6-o-o-diacetyl-britannilactone
ADC: Ajudecunoid C.
